# Examining the differential effects of information about the death penalty on retributivists and non-retributivists in Japan: a refutation of Marshall's third hypothesis

**DOI:** 10.3389/fpsyg.2023.1236587

**Published:** 2023-09-14

**Authors:** Eiichiro Watamura, Tomohiro Ioku, Tomoya Mukai

**Affiliations:** ^1^Graduate School of Human Sciences, Osaka University, Osaka, Japan; ^2^Graduate Schools for Law and Politics, University of Tokyo, Tokyo, Japan

**Keywords:** capital punishment, death penalty, deterrence, false convictions, Japanese public opinion, Marshall hypothesis

## Abstract

This study aimed to test Marshall's third hypothesis—that information about the death penalty hardly affects the attitude of death penalty supporters on retribution grounds—utilizing a non-American sample. Four pre-registered experiments were conducted, involving Japanese participants randomly selected from sample pools of retributivists and non-retributivists, based on their reasons for supporting the death penalty. One group received information exposure, while the other was under control conditions. Participants read about deterrence (Study 1) or false convictions (Study 2–4). Except for the results of Study 4, retributivists and non-retributivists were equally affected or unaffected by information. Marshall's third hypothesis is therefore not supported. Retributivists strongly favored the death penalty; higher empathy toward criminals was associated with less pro-death penalty attitudes. Additionally, there were differences in the influence of information. These results suggest the need for a new approach to researching the relationship between public attitudes and information on the death penalty.

## 1. Introduction

More than 50 years ago, Justice Marshall said: “If the American people knew all available information about the death penalty, they would not regard it as the best option” (Furman v. Georgia, 1972). Since then, Marshall's words have been subdivided and tested as three hypotheses (Cochran, 2017): (1) The general public supports the death penalty because they are ignorant of it; (2) Exposure to information about the death penalty makes them less likely to support it; and (3) However, information has little or no effect on those who support the death penalty on retribution grounds.

The first hypothesis refers to the correlation between the amount of information about the death penalty and an individual's support for it; the second refers to the causal effect of information on that support; and the third refers to an exception to the second hypothesis. This study aimed to confirm the third hypothesis, as other studies have tested the other two hypotheses (Lee et al., 2014). This hypothesis's truth helps us understand arguments that do not hold for some proponents. For example, the finding that the death penalty has no crime deterrent effect (Radelet and Akers, 1996; Muramatsu et al., 2018) is one of the grounds for abolition (Ellsworth and Ross, 1983); however, if the third hypothesis is supported, this finding would do little to advance the debate among proponents of retributivism, suggesting the need for placing a different topic on the table. This study also aimed to test the hypothesis on a non-US sample; of the G7 group of advanced democracies, only the US and Japan have the death penalty. Most experiments involving the Marshall hypothesis have been conducted in the US, and a few in Japan. Examining a Japanese sample would provide valuable clues to understanding the relationship between the death penalty and public opinion in democracies.

## 2. Literature review

The second hypothesis is reviewed before the third, which addresses the exceptions to the second hypothesis. The second hypothesis predicts that information about the death penalty will strengthen anti-death penalty attitudes among the general public. Previous research compared the participants' attitudes in the informed condition to those in the control condition. The information provided included essays denying the crime deterrent effect of the death penalty (Sarat and Vidmar, 1976; Lambert and Clarke, 2001; Lambert et al., 2011), stories about an unfortunate man who was falsely convicted and sentenced to death (Norris and Mullinix, 2020), the rate and number of wrongful convictions (Bobo and Johnson, 2004; Norris and Mullinix, 2020; Wu, 2021), the global trend of death penalty abolition (Lachappelle, 2014; Liang et al., 2019), humanitarian information on the psychological and physical effects of the death penalty on offenders (Sarat and Vidmar, 1976), and school courses that educate about the death penalty (Bohm, 1989; Bohm et al., 1991; Cochran et al., 2006; Kennedy-Kollar and Mandery, 2010; Lee et al., 2014; Harmon et al., 2021). The results showed that participants exposed to information tended to become less supportive of the death penalty—although not in all experiments.

Nevertheless, susceptibility to influence by information varies from person to person. According to the Elaboration Likelihood Model, the content of a persuasive message is not examined if the message does not affect the person (low personal relevance) or if the need for cognition is low (Petty et al., 1986; Wagner and Petty, 2011). Moreover, even when examined, the desire to be consistent in one's actions, statements, attitudes, and beliefs causes people to selectively pursue information consistent with their attitudes (i.e., cognitive consistency; Simon et al., 2004); thereby, they protect their identity by denying inconsistent information (Westerwick et al., 2013; Washburn and Skitka, 2018). Attitudes toward the legal system reflect an individual's views on justice (Vollum et al., 2004, 2009), and the death penalty symbolizes strong beliefs to counter moral threats (Vidmar and Ellsworth, 1974; Tyler and Weber, 1982; Lynch, 2002). The death penalty is considered by some to be a fair system based on retributive justice (Carlsmith, 2008), as it allows the perpetrator who took a human life to receive the same retribution (death). Therefore, some supporters who view the death penalty as just are unwilling to accept information that encourages disapproval (Lord et al., 1979). Importantly, there are various reasons why people support the death penalty, including retribution, deterrence, and incapacitation (Finckenauer, 1988; Jiang et al., 2010; Andreescu and Hughes, 2020; Griffin, 2021). Marshall's third hypothesis predicts that information will have a weaker effect on those who support the death penalty on retribution grounds than on those who support it for other reasons (i.e., it involves the interaction between information and reason). This is consistent with the assumption that people's punishment decisions are intuitively determined by retributive motives—rather than as a result of information deliberation—and are, therefore, difficult to modify based on information (Carlsmith, 2006, 2008).

However, only a few studies have tested the third hypothesis (Lee et al., 2014), and these have several limitations. The most assignable is the response–order effect (Israel and Taylor, 1990; Krosnick, 2018). In Ellsworth and Ross (1983) and Kimura (2015) experiments, when respondents were asked why they supported the death penalty—along with or immediately before the additional question, “Would you support the death penalty if it had no crime deterrent effect?”—the responses showed that retributivists displayed little change in attitude. However, it was unlikely that participants who had just chosen retribution would change their attitude to disapproval. In contrast, those who chose deterrence were under pressure to change their attitudes (i.e., demand characteristics). Lee et al. (2014) eliminated response bias using a Solomon four-group experimental design: two experimental groups were exposed to information, while two control groups were not. The pretest's effect was controlled for by having only one subgroup from each group (experiment/control) take the pretest. Results showed no difference in attitudes after information exposure between those with stronger and weaker retributivist tendencies; thus, Marshall's third hypothesis was not supported.

Although Lee et al. (2014) idea for removing response bias was groundbreaking, several problems remained. First, participants were not exclusively supporters of the death penalty. Given Marshall's statement that the attitudes of “supporters” of retributivism are less sensitive to information, the focus should have been solely on supporters. Second, they did not examine attitude change among the same participants. Their Solomon four-group experimental design involved comparisons between different groups; therefore, individual differences in knowledge about the death penalty could not be eliminated. Finally, the experimental results' generalizability was compromised, as their participants were college students taking a death penalty class, not a random sample from the general public. Apart from Lee et al. (2014) limitations, it is imperative to examine whether the study of public opinion on the death penalty can be replicated outside the US (Stack, 2004). Strong support for the death penalty in Japan is based on moral intuition (Johnson, 2020) and admitted ignorance about the death penalty (Kimura, 2015). Similar to the US, retribution and deterrence are the two most common reasons for supporting the death penalty (Cabinet Office Government of Japan, 2019). In addition to these common socio-psychological characteristics, the US and Japan are common in their political economies, both being democracies and G7 countries, and an examination of the two countries may elucidate the relationship between the death penalty and public opinion (Neumayer, 2008).

## 3. Present study

To recapitulate the limitations of Lee et al. (2014), an experiment must be conducted involving death penalty supporters who are randomly selected from the general public to examine Marshall's third hypothesis. To control for individual differences in knowledge about the death penalty, both a pretest (asking for reasons), and a posttest (asking for attitudes after information exposure), should be administered to the same individuals; however, response bias must be prevented during this process. The current study additionally aimed to validate the results with non-US participants. Therefore, we conducted a preliminary survey of randomly selected Japanese individuals 2 weeks before the main experiment. Based on the survey results, we selected participants who supported the death penalty on retribution grounds and those who supported it for other reasons. In each of the four experiments, participants drawn from the retributivist and non-retributivist sample pools were randomly assigned to an experimental group who were exposed to information, or a non-exposed control group. Once sampled, participants were excluded from the next experiment.

Although we cannot be certain, considering the lack of references, we determined that 2 weeks was long enough to prevent response bias and not too long to alter participants' attitudes or reasons. Three representative types of information were selected from previous studies for exposure to the experimental condition: Study 1 (*N* = 368) used an essay denying the crime deterrent effect of the death penalty; Study 2 (*N* = 368) used an essay describing an unfortunate man who was wrongly convicted; and Study 3 (*N* = 368) used the rate of false convictions for murder. In Study 4 (*N* = 736), we increased the rate of false convictions to four levels. To summarize, Studies 1 through 3 were 2 × 2, and Study 4 was a 2 × 4 between-participants design. The dependent variable across the four studies was the attitude toward the death penalty, and participants were asked to rate the extent to which they were pro- or anti-death penalty after information exposure. We predicted that non-retributivists would reduce their pro-death penalty attitudes, while retributivists' attitudes would not be affected to the same degree. The two hypotheses are as follows:

H1: Exposure to information about the death penalty reduces pro-death penalty attitudes.

H2: However, the effect of such information is weak on those who support the death penalty on retribution grounds.

Hypothesis 1 above corresponded to Marshall's second hypothesis and Hypothesis 2 to Marshall's third hypothesis, although the numbers are, confoundingly, off by one. Assuming that Hypothesis 1 was confirmed, we anticipated that its exception, Hypothesis 2, would be supported. This study was pre-registered. Our hypotheses, experimental design, and analytical design are registered in the Open Science Framework, from where all materials, including raw data and questions, can be downloaded. This research was conducted as part of the project “An Empirical Study on the Public's Judgment of Quantitative Sentencing,” which was reviewed by the Ethics Review Committee for Behavioral Sciences, Graduate School of Human Sciences, Osaka University (approval number: HB021-125).

## 4. Materials and methods

### 4.1. Preliminary survey

We randomly selected 87,699 Japanese individuals aged 18 or older from a panel of 13 million people registered with an Internet research firm in Japan, using the search function of MySQL, and sent them an invitation e-mail. The 8,000 people who provided informed consent (equal numbers of men and women, *M*_age_ = 42.8, *SD* = 12.8) were presented with the following one-item question and asked to choose one option.

*Please choose one option that best describes your view of the death penalty in Japan*.

1. *The death penalty should remain as a form of retribution, that is, a punishment for wrongdoing*.
2. *The death penalty should remain as a crime deterrent, that is, as a threat to someone who commits a crime*.
3. *The death penalty should remain in place for reasons other than these two*.
4. *The death penalty should be abolished*.
5. *None of the above apply*.

### 4.2. Study 1

Study 1 employed a 2 × 2 between-participants design, with the first independent variable being reasons in favor of the death penalty; this variable had two levels: retributivism and non-retributivism. The second independent variable was an essay. In the experimental condition, the essay used was a shortened version of the essay by Sarat and Vidmar (1976), which explained that the death penalty has no crime deterrent effect (Supplementary material). The original essay was ~1,500 words. Considering that online participants often experience low concentration (Alvarez et al., 2019), we shortened it to 464 words to encourage responses. The essay in the control condition was an introductory essay (488 words) on police organizations—and unrelated to the death penalty—taken from the Ministry of Justice website (Supplementary material). After reading each essay, we compared the two groups' attitudes toward the death penalty. Based on Hypotheses 1 and 2, we predicted the following:

*Attitudes in favor of the death penalty will reduce in the experimental condition*.

*There is a reason–essay interaction effect. The essay that denies the crime deterrence effect has a lesser effect on retributivists*.

#### 4.2.1. Participants

We first performed a power analysis, the results of which were f2 =0.15, α =0.05, and power (1-β) =0.95, indicating that 46 participants per group would be needed. We estimated that the total number of participants who did not provide informed consent or who would be excluded by the attention check (see analytic strategy) would be 50%, and we pre-registered the study for a target sampling of 92 participants in each group. Participants drawn from the retributivist pool were randomly allocated to either the experimental condition or the control condition, and two groups were also created from the non-retributivist pool (*N* = 368, [female = 153, male = 215], *M*_age_ = 44.0, *SD* = 12.1).

#### 4.2.2. Procedures

Of the 368 participants, 338 who provided written informed consent were directed to the experimental screen. They were first presented with one of two essays, after which they were asked about their attitudes toward the death penalty. To the question, “Do you agree or disagree with the death penalty for those convicted of murder?” they responded on a 4-point Likert-type scale ranging from 1 (“strongly agree”) to 4 (“strongly disagree”). The covariates were perceived risk of victimization, perceived risk of victimization (vicarious), fear of crime, perceived crime rate, empathy toward criminals, and empathy toward victims. These items and options were the same as those in the work of Wu (2021). Demographic characteristics such as gender, age, education, and income were also recorded.

#### 4.2.3. Analytic strategy

Before analysis, we excluded 43 participants who answered “don't know” to questions regarding attitudes toward the death penalty. We also excluded 59 participants who answered incorrectly to at least one of the two attention checks designed to exclude participants answering the questions without reading the questionnaire. In the end, 236 participants (female = 89, male = 147, *M*_age_ = 46.1, *SD* = 12.6) were included in the analysis of four groups, ranging from 51 to 67 participants per group. A multiple regression analysis was conducted using attitudes toward the death penalty as the dependent variable, reasons for supporting the death penalty and essays as independent variables, six covariates, and four demographic variables. The analysis was conducted using HAD software (Shimizu, 2016).

### 4.3. Study 2

The information provided in Study 2 was an essay about an unfortunate man who was incarcerated for 9 years because of a false arrest, taken from the work of Norris and Mullinix (2020). Their study confirmed that this essay reduced participants' support for the death penalty. We used a Japanese translation of this essay, with little change in content or length (326 words; Supplementary material). The essay for the control condition was the same police introduction as in Study 1. Study 2 also had a 2 × 2 between-participant design, combining a reason (retributivism, non-retributivism) and an essay (wrongful arrest essay, police introduction).

#### 4.3.1. Participants

Four groups of 92 participants each were created. Two groups were randomly selected from the retributivist pool and two groups from the non-retributivist pool (female = 183, male = 185, *M*_age_ = 44.7, *SD* = 12.7), after excluding the participants of Study 1. From the 347 participants who provided informed consent, ineligible participants were excluded using the same criteria as in Study 1, leaving a total of 236 participants (female = 85, male = 114, *M*_age_ = 47.5, *SD* = 12.8) in four groups for analysis.

### 4.4. Study 3

The essay information in Studies 1 and 2 was cited from previous studies and represented only a one-sided argument against the death penalty (Kennedy-Kollar and Mander 2010); moreover, whether it corresponds to Justice Marshall's intended meaning is controversial (Wu, 2021). The information used in Studies 3 and 4 was the rate of false convictions. In previous studies (Bobo and Johnson, 2004; Norris and Mullinix, 2020; Wu, 2021), participants who were presented with the rate of false convictions diminished their pro-death penalty attitudes; Study 3 used the information “the rate of false convictions for murder cases is 4.1%”. In Wu (2021) work, four levels (4.1%, 1%, 0.027%, and no information) were compared to detect the tipping point at which the participants switched between being in favor of and being against the death penalty. In that experiment, a clear effect at the 4.1% condition was observed. As the purpose of this study was to determine whether the effect of information differs depending on the reason, regardless of the tipping point, only two levels−4.1% and no information—were compared.

#### 4.4.1. Participants

After excluding participants from Studies 1 and 2, two groups of 92 participants each from the retributivist pool and the same from the non-retributivist pool were randomly selected (female = 178, male = 190, *M*_age_ = 44.1, *SD* = 12.8). A total of 247 participants (female = 121, male = 126, *M*_age_ = 44.7, *SD* = 12.9) who passed the same exclusion criteria as before were included in the analysis.

### 4.5. Study 4

According to Wu (2021), the higher the rate of false convictions, the stronger the effect on attitudes toward the death penalty, with the tipping point being between 1% and 4.1%. In the current study, the 4.1% rate was above the tipping point for most participants, which may explain why, in Study 3, even retributivists may have opposed the death penalty (see Section 5.4). Specifically, the low rates of false convictions that do not reach the tipping point may make a difference, for various reasons. To investigate this possibility, in Study 4, we compared the same four levels (4.1%, 1%, 0.027%, and no information) as Wu (2021). Study 4 was a 2 × 4 between-participants design comprising eight groups with a combination of reasons and four different rates of false convictions. At low rates of false convictions, only the non-retributivists were predicted to reduce pro-death penalty attitudes, while at high rates of false convictions, both retributivists and non-retributivists were predicted to be less supportive of the death penalty.

#### 4.5.1. Participants

After excluding all previous participants, four groups of 92 participants each from the retributivist pool and four groups of 92 participants each from the non-retributivist pool were randomly sampled (female = 355, male = 381, *M*_age_ = 44.5, *SD* = 12.7).

#### 4.5.2. Analytic strategy

The 454 participants (female = 206, male = 248, *M*_age_ = 44.8, *SD* = 13.2) who passed the exclusion criteria were included in the analysis. In Study 4, in addition to a multiple regression analysis with attitudes toward the death penalty as the dependent variable, we also calculated the probability of how well each rate of false conviction predicted attitudes (prediction margin), based on regression models, as in Wu (2021).

## 5. Results

### 5.1. Preliminary survey

The results of the preliminary study showed that (1) 3,562 people (44.5%) support the death penalty on retribution grounds, (2) 2,141 (26.8%) support it for crime deterrence, and (3) 963 (12.0%) support it for reasons other than those two. Group 1 was the retributivist pool, and Groups 2 and 3 were combined to form the non-retributivist pool (3,104 respondents). Note that both pools comprised supporters only. In the four subsequent experiments, participants were recruited from these two pools of supporters. Those who indicated that there were no options that should be repealed or that applied to them (1,334 respondents, 16.7%) were excluded from subsequent analyses.

### 5.2. Study 1

The descriptive statistics and partial correlation coefficients^1^ for Study 1 are shown in Supplementary Table 1. Higher values for attitudes toward the death penalty, measured using a 4-point scale, indicated less pro-death penalty attitudes; higher values for empathy toward criminals indicated less support for the death penalty (*r* = 0.33, *p* < 0.01), and higher values for fear of crime and empathy toward victims were associated with more support for the death penalty (*r* = −0.17, *p* < 0.05 and *r* = −0.17, *p* < 0.01, respectively). Women (coded as 0) tended to be less supportive of the death penalty (*r* = −0.21, *p* < 0.01).

A multiple regression analysis was conducted to test the hypotheses (Supplementary Table 2). The analysis did not confirm the main effect of the essay, contrary to our prediction (β = 0.06, *p* = 0.27). As for participants in the experimental condition who read the essay that negated the crime deterrent effect, the information did not reduce their pro-death penalty attitudes. No interaction between reason and essay was confirmed (β = 0.01, *p* = 0.88). While both predictions were not confirmed, the main effect of the reason was found. Retributivists (coded as 1) were more strongly in favor of the death penalty than non-retributivists (β = −0.21, *p* < 0.001). Higher empathy toward criminals was associated with less pro-death penalty attitudes (β = 0.28, *p* < 0.001), while those with higher empathy toward victims were more strongly in favor of the death penalty (β = −0.18, *p* < 0.01). Women also showed significantly less pro-death penalty attitudes (β = −0.22, *p* < 0.001).

Study 1 used an essay (Sarat and Vidmar, 1976) denying the crime deterrent effect of the death penalty to compare attitudes toward the death penalty of retributivists and non-retributivists, before and after information exposure. Linear regression modeling was used to test the effects of reason and essay. Our multiple regression analysis revealed neither an interaction of reason × essay, confirming Marshall's third hypothesis, nor a main effect of the essay implying its premise, Marshall's second hypothesis. Previous studies show that participants who read the essay strengthened their anti-death penalty attitudes (Sarat and Vidmar, 1976; Lambert and Clarke, 2001; Lambert et al., 2011); however, this did not occur in our experiment. The essay was shortened, which may have weakened the effect. Perhaps the Japanese participants were not persuaded because of the essay title: “American research shows no deterrent effect”. It is also possible that the essay was one-sided and did not include any positive information on the death penalty based on an anti-death penalty position, which could have created psychological reactance (Brehm, 1989; Rosenberg and Siegel, 2018). Most importantly, our participants were exclusively supporters of the death penalty. As deterrence is information that can justify their pro-death penalty attitudes (Wynarczyk, 1999; Galliher and Galliher, 2001), its denial may have proved resistant for supporters.

### 5.3. Study 2

Supplementary Table 3 shows the descriptive statistics and partial correlation coefficients for Study 2. As in Study 1, higher levels of empathy toward criminals were associated with less support for the death penalty (*r* = 0.40, *p* < 0.01). Moreover, higher income indicated a more positive attitude toward the death penalty (*r* = −0.21, *p* < 0.01).

In contrast to Study 1, Study 2—that used essays describing misfortune—confirmed the main effect of the essay (β = 0.24, *p* < 0.01), with participants who read the essays (coded as “1”) reducing their pro-death penalty attitudes. Our results are consistent with Norris and Mullinix (2020) explanation, that individual experiences are likely to influence attitudes. However, a reason × essay interaction was not confirmed (β =0.01, *p* =0.85). Consistent with Study 1, retributivists were as affected by the misfortune essay as non-retributivists, even though they were more strongly in favor of the death penalty (β = −0.13, *p* = 0.07). Reasons for supporting the death penalty were not investigated in Norris and Mullinix (2020). In our experiment, we tested both groups separately, using the same misfortune essay. In line with Study 1, there was a significant positive effect of empathy toward criminals (β = 0.36, *p* < 0.01), and higher empathy reduced pro-death penalty attitudes. The demographic variable that predicted attitudes in favor of the death penalty in Study 1 was gender (female <male), whereas in Study 2, the higher an individual's income, the more likely that individual was to be in favor of the death penalty (*r* = −0.18, *p* < 0.01; Supplementary Table 4).

### 5.4. Study 3

The descriptive statistics and partial correlation coefficients for Study 3 are shown in Supplementary Table 5. Consistent with the previous two studies, the higher the empathy toward criminals, the less pro-death penalty the participants are (*r* = 0.39, *p* < 0.01). Moreover, as in Study 1, women tended to be less supportive of the death penalty (*r* = −0.14, *p* = 0.03).

The results of the multiple regression analysis are shown in Supplementary Table 6. As predicted, the results showed that, when given the information that “the rate of false convictions is 4.1%,” the respondents showed weaker support for the death penalty (β = 0.19, *p* < 0.01). This result was consistent with that of previous studies (Bobo and Johnson, 2004; Norris and Mullinix, 2020; Wu, 2021) and supported Marshall's second hypothesis. However, the exception to the second hypothesis, that is, the third hypothesis—the reason × rate of false convictions interaction—was again non-significant (β = 0.09, *p* = 0.11) and therefore not supported, even by changing the information from the essay format to rate of false convictions. As 4.1% was a high rate of false convictions, it may have influenced even retributivists. As in Studies 1 and 2, a main effect of the reason was confirmed (β = −0.16, *p* < 0.01). Retributivists were also more in favor of the death penalty than non-retributivists, again confirming the relationship between empathy toward criminals and attitudes toward the death penalty (β = −0.37, *p* < 0.01). Women were less supportive of the death penalty (β = −0.14, *p* = 0.02).

### 5.5. Study 4

The partial correlation coefficients for Study 4, as shown in Supplementary Table 7, were consistent with the previous three studies, indicating that when empathy toward criminals was higher, the participants' support for the death penalty was less (*r* = 0.32, *p* < 0.01). The finding of women being less in favor of the death penalty (*r* = −0.13, *p* < 0.01) was similar to Studies 1 and 3.

A hierarchical multiple regression analysis was conducted to determine how much the rate of false convictions and reasons affect attitudes toward the death penalty. The reference group was the one with no information on the rate of false convictions (and was therefore excluded from the regression equation). In Step 1, no interaction term was entered. In Step 2, reason × rate of false conviction of 0.027%, in Step 3, reason × rate of false conviction of 1%, and in Step 4, reason × rate of false conviction of 4.1% were entered as independent variables. As the model's predictive power did not improve after Step 2 (R_2_s = 0.218), the Step 2 model, the simplest, was used (Supplementary Table 8; see also models for each Step uploaded to Open Science Framework).

The results of Study 4 were contrary to expectations: participants who were presented with rates of false convictions as high as 1% or 4.1% expressed stronger pro-death penalty attitudes (β = −0.22, *p* < 0.01; β = −0.13, *p* = 0.02, respectively). In Study 3, the participants who were presented with the false conviction rate of 4.1%, with the same procedure, expressed less favorable attitudes. Notably, consistent with Studies 1 through 3, the main effect of the reason was marginally significant in Study 4 (β = −0.07, *p* = 0.08), with retributivists more in favor of the death penalty than non-retributivists. The higher the empathy toward criminals, the less pro-death penalty the participants were (β = −0.30, *p* < 0.01), similar to the other three studies. Among the demographic variables, a significant trend was confirmed between age and pro-death penalty attitude (β = −0.08, *p* = 0.07).

We examined the predictive margin (Supplementary Figure 1) and discovered that the higher the false conviction rate, the stronger the attitude in favor of the death penalty. Support for the death penalty among non-retributivists increased progressively from 60% in the no information group to 66% in the 0.027% group, 71% in the 1% group, and 75% in the 4.1% group. Support for the death penalty from the retributivists was high, even in the no information group (79%); moreover, these rates increased further as the rate of false convictions increased, reaching 86% in the 1% group and 88% in the 4.1% group.

As shown in Supplementary Figure 2, an interaction between reason × 0.027% rate of false conviction (β = −0.09, *p* = 0.05) was confirmed. Non-retributivists presented with the information that “the rate of false convictions is 0.027%” weakened their pro-death penalty attitudes more than the retributivists, which suggests that the effect of the information on retributivists was limited only for the 0.027% condition.

## 6. Discussion

The results of our four pre-registered experiments involving a Japanese population who support the death penalty showed little difference in response to information on the death penalty between retributivists and non-retributivists. In Study 1, the essay denying the deterrent effect of the death penalty did not affect either group; when exposed to the misfortune essay in Study 2 and the rate of false convictions of 4.1% in Study 3, both groups demonstrated a reduction in pro-death penalty attitudes. Contrary to Study 3, Study 4 confirmed the trend of a higher rate of false convictions strengthening support for the death penalty, with an interaction effect of a false conviction rate of 0.027% only weakening pro-death penalty attitudes of non-retributivists. Thus, our results confirm that, (1) contrary to Marshall's second hypothesis, information about the death penalty may not affect attitudes when tested only among death penalty supporters, and (2) contrary to Marshall's third hypothesis, there is little difference in the effect of information on participants' attitudes related to different reasons for supporting the death penalty. These results are largely consistent with those of Lee et al. (2014), which involved a sample of American participants. Although the information and experimental designs differ between Lee et al. (2014) study and ours, the series of tests conducted to date, including the present study, indicate that Marshall's third hypothesis is not supported in either US or Japanese samples.

### 6.1. Theoretical implications

Across the four pre-registered experiments, few significant differences were found by reason for supporting the death penalty. In democracies that currently have the death penalty, such as the US and Japan, differences in reasons for support appear to be unrelated to the attitudinal effects of the information available.

Notably, we found differences in reasons for support in the strength of attitudes toward the death penalty rather than in susceptibility to information. The main effects of reasons were significant or close to significant consistently, across the four experiments. Among supporters, those who favor retribution are more strongly in favor of the death penalty. Another notable finding is that empathy toward criminals consistently decreased support. These two findings were common to all four studies and are, therefore, highly plausible. Individual differences in the effects of information, as suggested by Marshall's third hypothesis, should be reexamined with other approaches, such as commitment to attitudes toward the death penalty, which is more pronounced among retributivists, and also perspectives toward criminals.

We also found differences in the influence of information on attitudes toward the death penalty. Comparing the four studies, the information most likely to decrease support for the death penalty was the criminals' misfortune essay in Study 2, as indicated by the robust finding that empathy toward criminals weakened pro-death penalty attitudes in all four experiments. The misfortune episode of the falsely accused man may have made participants think “if I had been in the same situation…”; based on the Elaboration Likelihood Model, their pro-death penalty attitudes may have been affected because the information was more personally relevant, as participants were more likely to consider it in their own position. The effect of the rate of false convictions cannot be determined because the results of Studies 3 and 4 did not match. Moreover, essays denying the deterrent effect of crime in Study 1 had little effect. These differences in information could not be inferred from comparisons between previous studies, because of differences in factors other than information (e.g., demographic characteristics of the participants or question items). In our study, however, they could be clarified because they were almost the same across the four studies.

The likelihood of generating psychological reactance (Brehm, 1989; Rosenberg and Siegel, 2018) among death penalty supporters exposed to information was also shown. In Study 1, supporters who read the essay denying the deterrent effect of the death penalty remained in favor of the death penalty, while Study 4 participants showed more favorable attitudes as the rate of false conviction increased. The consistency of increased support with a higher rate of false convictions indicates the robustness of Study 4. One possible explanation of this counterintuitive increase is due to the boomerang effect, in which attempts to persuade an opponent are counterproductive, causing the opponent to adopt attitudes and behaviors opposite to the persuader's intentions. This effect is often seen in persuasions that deny identity-related beliefs and values (Byrne and Hart, 2009; Ma et al., 2019). As the death penalty is a symbol of a strong belief in countering moral threats for death penalty supporters (Vidmar and Ellsworth, 1974; Tyler and Weber, 1982; Lynch, 2002), they may have felt stronger opposition to the higher rate of false conviction. Presented with a higher false conviction rate, they may have thought, “This information is going to change my pro-death penalty attitude, but I will not go along with that intention; the death penalty is absolutely necessary”. In previous studies, while some supporters reacted, disapprovers and those who were impartial opposed the death penalty; therefore, information was more likely to influence participants.

### 6.2. Practical implications

Our results reiterate that information should be cautiously treated in future debates on the death penalty. Contrary to previous assumptions, some information may not have the intended impact. Although the lack of crime deterrent effect of the death penalty is cited as a rationale for abolition (Ellsworth and Ross, 1983), the results of this study suggest that it has little persuasive effect on supporters. Moreover, a high rate of false convictions may lead to reactance, further reinforcing their pro-death penalty attitudes, in a boomerang effect. The impetus for reconsideration among proponents suggested by this study is the public disclosure of the individual tragedies of false convictions. Another notable factor in deepening the debate on the death penalty policy is the main effect of reason. As retributivists were more consistently in favor of the death penalty than non-retributivists, those who value retributive justice (Carlsmith, 2006, 2008) may believe that the life of the victim can only be offset by the life of the perpetrator. The issue of whether the death penalty is the only way to achieve justice may be of interest to them. Knowing that their own beliefs are not dominant causes people to focus on the information they had previously ignored (Hall and Raimi, 2018). Once they realize that the death penalty is not the only option, information may become influential. The necessity of investigating attitudes toward the death penalty when selecting jurors and judges was also confirmed. Supporters are more likely to judge the death penalty in actual trials (Eisenberg et al., 2001; O'Neil et al., 2004). However, if attitudes were extremely unlikely to be affected by information, the significance of the jury panel would be diminished. Attitudes toward the death penalty and the reasons behind them might be applied as a selection criterion for determining whether prospective jurors can consider the information they receive in their deliberations.

### 6.3. Limitations and future research

Participants' reasons for supporting the death penalty may not have been accurately captured in the preliminary survey. Deterrence may be used as *post hoc* justification, rather than the real reason for punishment decisions (Carlsmith, 2006, 2008). The difference in reasons may not have been identified because participants who chose deterrence, when the real reason was retribution, were included in the pool of non-retributivists. Some participants may have placed equal importance on retribution and other reasons, such as deterrence. Although the preliminary survey instructed participants to choose one reason, it might have been better to let them assign a score for each reason. This would have enabled us to select pure retributivists with high retribution scores only. Moreover, the response bias may not have disappeared in the 2 weeks between the preliminary survey and the main experiment. However, had the period been too long, attitudes and reasons may have changed during that time. The appropriate length of time to eliminate response bias must be examined in the future. For example, one method would be to examine how long it takes for the difference in the impact of the information to disappear in the group that is asked about reasons and the group that is not. After the main experiment, respondents could be asked how aware they were of their preliminary survey responses. Finally, a limitation related to the experimental methodology is the absence of manipulation checks. For example, the manipulation of false conviction rates in Studies 3 and 4 required us to check how high participants rated each level of false conviction rates. By examining the relationship between the rating scores and attitudes toward the death penalty, we could have verified whether a higher false conviction rate (even for the same false conviction rate) makes the participants feel less supportive of the death penalty. It would have also provided insight into the discrepancy between Study 3 and Study 4 findings.

### 6.4. Conclusions

In this study, Marshall's third hypothesis was not confirmed. We found that, as in the US, information about the death penalty had approximately the same effect on supporters, regardless of the reason. Two additional novel and robust findings were that differences in reasons were evident in the strength of attitudes in favor of the death penalty, and that empathy for the criminals reduced support for the death penalty. These two findings and the different effects of different types of information may provide a new approach to elucidating the relationship between attitudes toward the death penalty and information regarding it in future research.

## Data availability statement

The datasets presented in this study can be found in online repositories. The datasets generated for this study can be found in the Open Science Framework, https://doi.org/10.17605/OSF.IO/J7EYT.

## Ethics statement

The studies involving humans were approved by the Ethical Review Committee for Behavioral Sciences, Graduate School of Human Sciences, Osaka University. The studies were conducted in accordance with the local legislation and institutional requirements. The participants provided their written informed consent to participate in this study.

## Author contributions

EW: supervision, conceptualization, methodology, writing—original draft, and funding acquisition. TI: formal analysis and writing—review and editing. TM: conceptualization and methodology—review and editing. All authors contributed to the article and approved the submitted version.

## Footnotes

^1^Considering the relationship between each variable and attitudes toward the death penalty could have been influenced by other variables, we examined partial correlations excluding other variables, rather than zero-order correlations.
