# Supplementary material for: Examining the differential effects of information about the death penalty on retributivists and non-retributivists in Japan: a refutation of Marshall's third hypothesis
Source: Front Psychol. 2023 Sep 14;14:1236587. doi: 10.3389/fpsyg.2023.1236587 (PMC10538536; doi:10.3389/fpsyg.2023.1236587)
Supplement: Supplementary file 1 [file Data_Sheet_1.docx]

Supplementary Material

Examining the Differential Effects of Information about the Death Penalty on Retributivists and Non-Retributivists in Japan: A Refutation of Marshall's Third Hypothesis

Eiichiro Watamura^*^, Tomohiro Ioku, Tomoya Mukai

*** Correspondence:** Eiichiro Watamura: watamura@hus.osaka-u.ac.jp

# Supplementary Figures and Tables

## Supplementary Figures

**Supplementary Figure 1.** Predicted probability of death penalty support conditional on different rate of false convictions.

**Supplementary Figure 2.** Attitudes toward the death penalty in the 0.027% rate of false conviction condition. Higher scores indicate greater opposition to the death penalty. Error bars represent standard errors.

## Supplementary Tables

**Supplementary Table 1.** Descriptive statistics and partial correlation coefficients for each variable in Study 1. **p* < .05; ***p* < .01.

|  |  | *M* | *SD* | 1 | 2 | 3 | 4 | 5 | 6 | 7 | 8 | 9 | 10 | 11 |
| --- | --- | --- | --- | --- | --- | --- | --- | --- | --- | --- | --- | --- | --- | --- |
| 1 | Attitude toward the death penalty | 1.77 | 0.56 | - | .00 | .06 | -.17* | -.10 | .33** | -.17** | .02 | -.21** | .06 | -.11 |
| 2 | Perceived risk of victimization | 2.32 | 1.01 |  | - | .81** | -.06 | .16* | .04 | .00 | .09 | .02 | -.08 | .09 |
| 3 | Perceived risk of victimization (vicarious) | 2.28 | 0.99 |  |  | - | .05 | -.08 | -.06 | .01 | -.06 | -.01 | .01 | -.06 |
| 4 | Fear of crime | 4.32 | 0.72 |  |  |  | - | .04 | -.10 | .01 | .04 | -.34** | -.04 | .05 |
| 5 | Perceived crime rate | 1.72 | 0.64 |  |  |  |  | - | -.04 | -.11 | -.14* | -.03 | -.03 | -.03 |
| 6 | Empathy toward criminals | 2.42 | 0.74 |  |  |  |  |  | - | .07 | .06 | -.10 | .05 | -.06 |
| 7 | Empathy toward victims | 4.08 | 0.72 |  |  |  |  |  |  | - | .13* | -.13* | .10 | -.04 |
| 8 | Age | 46.06 | 12.59 |  |  |  |  |  |  |  | - | .31** | -.06 | .01 |
| 9 | Gender (0 = Female) | 0.62 | 0.49 |  |  |  |  |  |  |  |  | - | .10 | .04 |
| 10 | Education | 3.81 | 1.44 |  |  |  |  |  |  |  |  |  | - | .26** |
| 11 | Income | 5.50 | 2.84 |  |  |  |  |  |  |  |  |  |  | - |

**Supplementary Table 2.** Multiple regression analysis of essays on attitudes against the death penalty. Coefficients represent standardized coefficients, CI: confidence interval, VIF: variance inflation factor. ** *p* < .01, * *p* < .05.

| **Independent Variable** | *β* |  | 95% CI | | VIF |
| --- | --- | --- | --- | --- | --- |
|  |  |  | Min | Max |  |
| Reason (0 = non-retributivist) | -.21 | ** | -.33 | -.09 | 1.09 |
| Essay (0 = control essay) | .06 |  | -.05 | .18 | 1.03 |
| Reason*Essay | .01 |  | -.10 | .12 | 1.03 |
| Perceived risk of victimization | .01 |  | -.19 | .20 | 3.04 |
| Perceived risk of victimization (vicarious) | .10 |  | -.10 | .29 | 2.97 |
| Fear of crime | -.15 | * | -.27 | -.03 | 1.16 |
| Perceived crime rate | -.09 |  | -.20 | .03 | 1.09 |
| Empathy toward criminals | .28 | ** | .16 | .40 | 1.11 |
| Empathy toward victims | -.18 | ** | -.30 | -.07 | 1.08 |
| Age | .01 |  | -.11 | .13 | 1.18 |
| Gender | -.22 | ** | -.35 | -.10 | 1.31 |
| Education | .02 |  | -.10 | .14 | 1.14 |
| Income | -.08 |  | -.20 | .04 | 1.11 |
|  |  |  |  | | |
| *R*^2^ | .28 | ** |  | | |
| Adjust *R*^2^ | .24 | ** |  |  |  |
| *F* | 6.77 |  | AIC | 346.08 |  |
| df | 13, 222 |  | BIC | 398.04 |  |
| *p* | .00 |  |  |  |  |
|  | | | | | |

**Supplementary Table 3.** Descriptive statistics and partial correlation coefficients for each variable in Study 2. **p* < .05; ***p* < .01.

|  |  | *M* | *SD* | 1 | 2 | 3 | 4 | 5 | 6 | 7 | 8 | 9 | 10 | 11 |
| --- | --- | --- | --- | --- | --- | --- | --- | --- | --- | --- | --- | --- | --- | --- |
| 1 | Attitudes toward the death penalty | 1.91 | 0.61 | - | -.05 | .13 | -.03 | .01 | .40** | .04 | .05 | -.01 | .03 | -.17* |
| 2 | Perceived risk of victimization | 2.31 | 1.00 |  | - | .82** | -.02 | -.06 | .12 | .24** | -.11 | -.02 | -.20** | -.13 |
| 3 | Perceived risk of victimization (vicarious) | 2.32 | 0.99 |  |  | - | -.03 | .07 | -.03 | -.19** | .07 | -.05 | .12 | .13 |
| 4 | Fear of crime | 4.34 | 0.73 |  |  |  | - | .08 | .00 | .12 | .03 | -.31** | -.05 | .03 |
| 5 | Perceived crime rate | 1.66 | 0.62 |  |  |  |  | - | -.12 | -.07 | -.13 | .00 | .01 | -.15* |
| 6 | Empathy toward criminals | 2.55 | 0.71 |  |  |  |  |  | - | -.15* | -.03 | .03 | .14 | .03 |
| 7 | Empathy toward victims | 4.23 | 0.70 |  |  |  |  |  |  | - | .26** | .03 | .06 | .10 |
| 8 | Age | 47.48 | 12.81 |  |  |  |  |  |  |  | - | .33** | -.01 | -.11 |
| 9 | Gender (0 = Female) | 0.57 | 0.50 |  |  |  |  |  |  |  |  | - | .15* | .03 |
| 10 | Education | 3.90 | 1.44 |  |  |  |  |  |  |  |  |  | - | .17* |
| 11 | Income | 5.67 | 2.98 |  |  |  |  |  |  |  |  |  |  | - |

**Supplementary Table 4.** Multiple regression analysis of essays on attitudes against the death penalty. Coefficients represent standardized coefficients, CI: confidence interval, VIF: variance inflation factor. ** *p* < .01, * *p* < .05, ^+^ *p* < .10.

| **Independent Variable** | *β* |  | 95% CI | | VIF |
| --- | --- | --- | --- | --- | --- |
|  |  |  | Min | Max |  |
| Reason (0 = non-retributivist) | -.13 | + | -.26 | .01 | 1.25 |
| Essay (0 = control essay) | .24 | ** | .11 | .36 | 1.06 |
| Reason*Essay | .01 |  | -.11 | .14 | 1.03 |
| Perceived risk of victimization | -.10 |  | -.32 | .13 | 3.40 |
| Perceived risk of victimization (vicarious) | .18 |  | -.04 | .40 | 3.24 |
| Fear of crime | .00 |  | -.13 | .13 | 1.17 |
| Perceived crime rate | .01 |  | -.12 | .13 | 1.09 |
| Empathy toward criminals | .36 | ** | .22 | .50 | 1.26 |
| Empathy toward victims | .05 |  | -.09 | .18 | 1.23 |
| Age | .03 |  | -.11 | .16 | 1.31 |
| Gender | .01 |  | -.13 | .15 | 1.36 |
| Education | -.02 |  | -.15 | .12 | 1.20 |
| Income | -.18 | ** | -.31 | -.05 | 1.10 |
| *R*^2^ | .29 | ** |  | | |
| Adjust *R*^2^ | .24 | ** |  |  |  |
| *F* | 5.90 |  | AIC | 327.66 |  |
| df | 13, 185 |  | BIC | 377.06 |  |
| *p* | .00 |  |  |  |  |
|  | | | | | |

**Table 5.** Descriptive statistics and partial correlation coefficients for each variable in Study 3. **p* < .05; ***p* < .01.

|  |  | *M* | *SD* | 1 | 2 | 3 | 4 | 5 | 6 | 7 | 8 | 9 | 10 | 11 |
| --- | --- | --- | --- | --- | --- | --- | --- | --- | --- | --- | --- | --- | --- | --- |
| 1 | Attitudes toward the death penalty | 1.81 | 0.56 | - | -.03 | -.01 | .00 | .03 | .39** | -.05 | -.10 | -.14* | .00 | -.03 |
| 2 | Perceived risk of victimization | 2.15 | 0.94 |  | - | .77** | .02 | .12 | .04 | -.02 | -.06 | .08 | -.06 | .00 |
| 3 | Perceived risk of victimization (vicarious) | 2.13 | 0.97 |  |  | - | .07 | .02 | .01 | .02 | .03 | -.06 | .01 | .00 |
| 4 | Fear of crime | 4.30 | 0.77 |  |  |  | - | .06 | -.03 | .18** | -.13* | -.20** | .04 | .02 |
| 5 | Perceived crime rate | 1.69 | 0.66 |  |  |  |  | - | -.06 | -.03 | -.03 | .02 | .02 | .04 |
| 6 | Empathy toward criminals | 2.51 | 0.74 |  |  |  |  |  | - | .01 | .14* | -.06 | .02 | -.10 |
| 7 | Empathy toward victims | 4.14 | 0.69 |  |  |  |  |  |  | - | .09 | .01 | .11 | .06 |
| 8 | Age | 44.70 | 12.86 |  |  |  |  |  |  |  | - | .15* | -.06 | -.10 |
| 9 | Gender (0 = Female) | 0.51 | 0.50 |  |  |  |  |  |  |  |  | - | .12 | .01 |
| 10 | Education | 3.79 | 1.50 |  |  |  |  |  |  |  |  |  | - | .16* |
| 11 | Income | 5.46 | 2.73 |  |  |  |  |  |  |  |  |  |  | - |

**Table 6.** Multiple regression analysis of rate of false conviction on attitudes against death penalty. Coefficients represent standardized coefficients, CI: confidence interval, VIF: variance inflation factor. ** *p* < .01, * *p* < .05, ^+^ *p* < .10.

| **Independent Variable** | β |  | 95% CI | | VIF |
| --- | --- | --- | --- | --- | --- |
|  |  |  | Min | Max |  |
| Reason (0 = non-retributivist) | -.16 | ** | -.28 | -.05 | 1.10 |
| False conviction (0 = no information) | .19 | ** | .08 | .30 | 1.02 |
| Reason*False conviction | .09 |  | -.02 | .20 | 1.02 |
| Perceived risk of victimization | -.07 |  | -.26 | .11 | 2.71 |
| Perceived risk of victimization (vicarious) | -.01 |  | -.19 | .17 | 2.66 |
| Fear of crime | .01 |  | -.11 | .13 | 1.15 |
| Perceived crime rate | -.02 |  | -.14 | .10 | 1.12 |
| Empathy toward criminals | .37 | ** | .25 | .49 | 1.10 |
| Empathy toward victims | -.03 |  | -.14 | .09 | 1.07 |
| Age | -.11 | + | -.23 | .00 | 1.11 |
| Gender | -.14 | * | -.26 | -.02 | 1.12 |
| Education | .00 |  | -.11 | .12 | 1.07 |
| Income | -.03 |  | -.15 | .08 | 1.08 |
| *R*^2^ | .29 | ** |  | | |
| Adjust *R*^2^ | .24 | ** |  |  |  |
| *F* | 6.35 |  | AIC | 365.12 |  |
| df | 13, 233 |  | BIC | 417.76 |  |
| *p* | .00 |  |  |  |  |
|  | | | | | |

**Table 7.** Descriptive statistics and partial correlation coefficients for each variable in Study 4. **p* < .05; ***p* < .01.

|  |  | *M* | *SD* | 1 | 2 | 3 | 4 | 5 | 6 | 7 | 8 | 9 | 10 | 11 |
| --- | --- | --- | --- | --- | --- | --- | --- | --- | --- | --- | --- | --- | --- | --- |
| 1 | Attitudes toward the death penalty | 2.08 | 0.73 | - | .04 | .04 | .00 | -.03 | .32** | .05 | .09 | -.13** | .01 | -.06 |
| 2 | Perceived risk of victimization | 2.23 | 0.97 |  | - | .77** | .01 | .05 | .00 | .01 | -.02 | -.01 | -.02 | .01 |
| 3 | Perceived risk of victimization (vicarious) | 2.22 | 1.00 |  |  | - | .03 | .07 | .02 | .00 | -.02 | .05 | .01 | .02 |
| 4 | Fear of crime | 4.31 | 0.71 |  |  |  | - | .03 | -.06 | .26** | -.09 | -.22** | .02 | .01 |
| 5 | Perceived crime rate | 1.72 | 0.63 |  |  |  |  | - | -.05 | -.02 | -.08 | -.04 | -.08 | -.05 |
| 6 | Empathy toward criminals | 2.41 | 0.70 |  |  |  |  |  | - | .07 | .07 | -.11* | .05 | -.07 |
| 7 | Empathy toward victims | 4.01 | 0.76 |  |  |  |  |  |  | - | .06 | -.01 | .03 | .11* |
| 8 | Age | 44.85 | 13.16 |  |  |  |  |  |  |  | - | .23** | -.08 | .03 |
| 9 | Gender (0 = Female) | 0.55 | 0.50 |  |  |  |  |  |  |  |  | - | .14** | .03 |
| 10 | Education | 3.76 | 1.44 |  |  |  |  |  |  |  |  |  | - | .25** |
| 11 | Income | 5.13 | 2.86 |  |  |  |  |  |  |  |  |  |  | - |

**Supplementary Table 8.** Multiple regression analysis of rate of false convictions on attitudes against the death penalty. This model was adopted as a result of the hierarchical multiple regression analysis. Coefficients represent standardized coefficients, CI: confidence interval, VIF: variance inflation factor. ** *p* < .01, * *p* < .05, ^+^ *p* < .10.

| **Independent Variable** | *β* |  | 95% CI | | VIF |
| --- | --- | --- | --- | --- | --- |
|  |  |  | Min | Max |  |
| Reason (0 = non-retributivist) | -.07 | + | -.16 | .01 | 1.04 |
| False conviction (1 = 0.027%) | .03 |  | -.07 | .13 | 1.48 |
| False conviction (1 = 1%) | -.22 | ** | -.33 | -.11 | 1.70 |
| False conviction (1 = 4.1%) | -.13 | * | -.24 | -.02 | 1.61 |
| Reason*False conviction 0.027% | -.09 | * | -.17 | .00 | 1.05 |
| Perceived risk of victimization | .07 |  | -.07 | .20 | 2.64 |
| Perceived risk of victimization (vicarious) | .03 |  | -.11 | .16 | 2.66 |
| Fear of crime | -.02 |  | -.11 | .07 | 1.18 |
| Perceived crime rate | -.03 |  | -.11 | .06 | 1.07 |
| Empathy toward criminals | .30 | ** | .21 | .39 | 1.09 |
| Empathy toward victims | .05 |  | -.03 | .14 | 1.11 |
| Age | .08 | + | -.01 | .17 | 1.10 |
| Gender | -.04 |  | -.13 | .06 | 1.39 |
| Education | .02 |  | -.07 | .11 | 1.12 |
| Income | -.06 |  | -.15 | .02 | 1.12 |
| *R*^2^ | .22 | ** |  | | |
| Adjust *R*^2^ | .19 | ** |  |  |  |
| *F* | 8.12 |  | AIC | 929.09 |  |
| df | 15, 438 |  | BIC | 999.10 |  |
| *p* | .00 |  |  |  |  |
